# Suicide risk after discharge from in-patient psychiatric care: A 15-year retrospective cohort study of individual patient data

**DOI:** 10.1192/j.eurpsy.2025.517

**Published:** 2025-08-26

**Authors:** A. Trojer, D. König-Castillo, A. Gleiss, B. Vyssoki, M. Grömer, S. Weber, A. Glahn, L. Sommer, C. Harrer, S. Listabarth, A. Wippel

**Affiliations:** 1 Clinical Division of Social Psychiatry, Department of Psychiatry and Psychotherapy; 2 Institute of Clinical Biometrics, Center for Medical Data Science, Medical University of Vienna, Vienna, Austria; 3 Department for Psychiatry, Social Psychiatry and Psychotherapy, Medical University of Hannover, Hannover, Germany

## Abstract

**Introduction:**

Suicide remains a leading cause of death worldwide, representing a significant public mental health challenge across all populations. Moreover, suicide rates are notably higher in patients following discharge from inpatient psychiatric care. Existing evidence regarding the specific risk factors for suicide in this population, however, remains contradictory. This study aims to systematically investigate those risk factors for post-discharge suicide death from a large psychiatric care facility.

**Objectives:**

To identify the risk factors associated with suicide following discharge from psychiatric care.

**Methods:**

Data from a 15-year single-centre cohort study were linked with national death registry records. Competing risk models were employed to calculate cumulative incidence rates. Key variables analyzed included sex, age at admission, discharge diagnosis, year of admission and length of stay. Subdistribution hazard ratios for these factors were computed using Fine-Gray models.

**Results:**

In the sample of 18,425 discharges from 10,973 individual patients (57.03% female), a relative excess hazard to die by suicide of 1.4 additional suicides per 100,000 population the first year after discharge compared to the general population was found. That risk of suicide after discharge was significantly higher for males (SHR = 1.67; p = 0.037) as well as for patients diagnosed with affective disorders (SHR = 3.56; p = 0.017) and neurotic stress and somatoform disorders (SHR = 3.73; p = 0.024). The risk of suicide decreased significantly in more recent discharge periods (SHR = 0.93; p = 0.006). The length of hospital stay did not show a statistically significant association with suicide risk (SHR = 0.98; p = 0.834).

**Image 1:**

**Figure:**
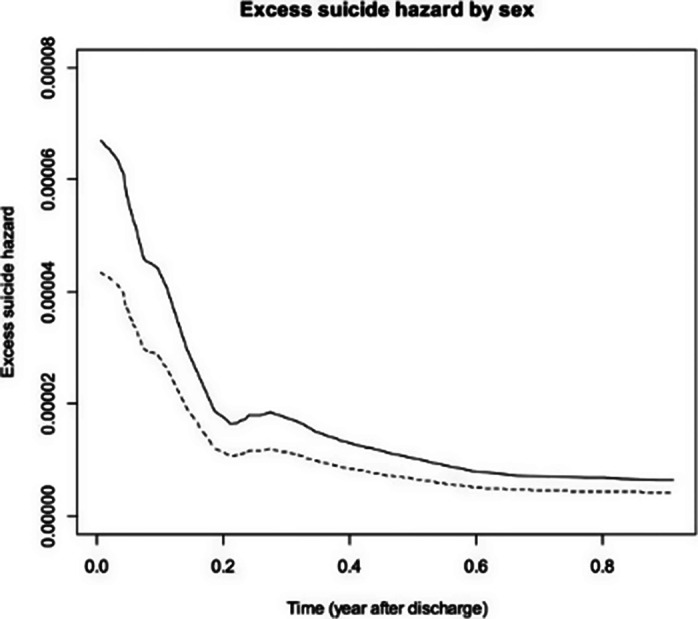

**Image 2:**

**Figure:**
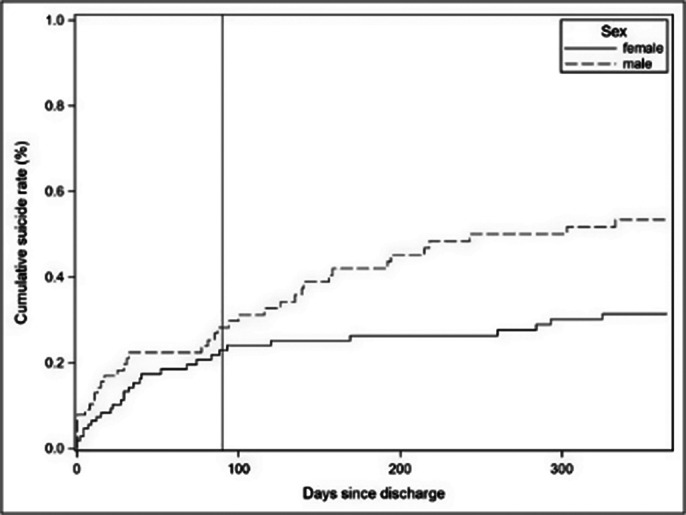

**Image 3:**

**Figure:**
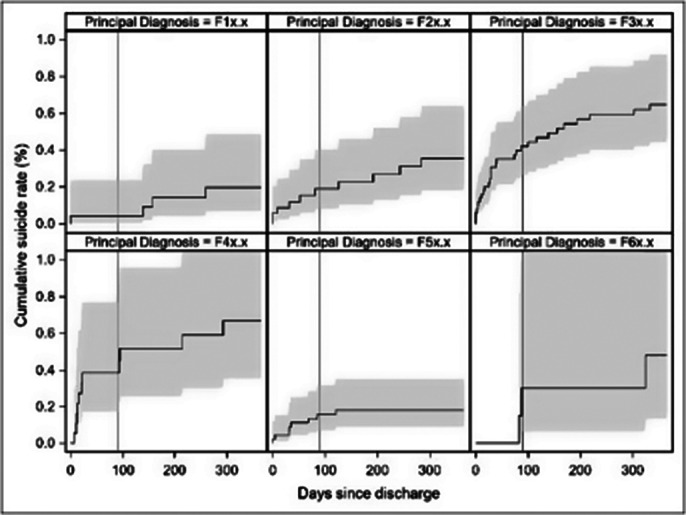

**Conclusions:**

Male sex and specific diagnoses, notably affective and neurotic stress and somatoform disorders, were associated with an increased risk of suicide following discharge from psychiatric care. The observed decrease in risk over time could potentially point to improvements in post-discharge patient management and or treatment. These findings underscore the necessity for enhanced risk assessment tools and targeted interventions to address suicide risk in discharged psychiatric patients.

**Disclosure of Interest:**

None Declared

